# Quality Control and Authentication of Black Betel Leaf Extract (*Piper acre* Blume) from East Kalimantan as an Antimicrobial Agent Using a Combination of High-Performance Liquid Chromatography and Chemometric Fourier Transform Infrared

**DOI:** 10.3390/molecules28155666

**Published:** 2023-07-26

**Authors:** Vina Maulidya, Aliya Nur Hasanah, Laode Rijai, Muchtaridi Muchtaridi

**Affiliations:** 1 Department of Pharmaceutical Analysis and Medicinal Chemistry, Faculty of Pharmacy, Universitas Padjajaran, Jl. Raya Jatinangor Km 21.5 Bandung-Sumedang, Bandung 45363, Indonesia; vimy23@gmail.com (V.M.); aliya.n.hasanah@unpad.ac.id (A.N.H.); 2 Faculty of Pharmacy, Universitas Mulawarman, Samarinda 75119, Indonesia; najwankhanrjai@yahoo.co.id; 3 Research Collaboration Center for Theranostic Radiopharmaceuticals, Jl. Raya Jatinangor Km 21.5 Bandung-Sumedang, Bandung 45363, Indonesia

**Keywords:** ethanol extract, black betel, piperenamide A, HPLC, FTIR, PCA, PLS-DA

## Abstract

Black betel leaf from East Kalimantan contains various secondary metabolites such as alkaloid saponins, flavonoids, and tannins. A compound, piperenamide A, which has antimicrobial activity, is also found in black betel leaf. This study aims to identify and authenticate the compound piperenamide A found in black betel leaf extract in other types of betel plant using HPLC and FTIR-chemometrics. The extraction method used was maceration with 70% ethanol solvent. Determination of piperenamide A content in black betel leaf extract was via HPLC column C18, with a maximum wavelength of 259 nm and a mobile phase of water:acetonitrile at a flow rate of 1 mL/minute. From the results, piperenamide A was only found in black betel (*Piper acre*) and not in *Piper betel* and *Piper crocatum*. Piperenamide A levels obtained were 4.03, 6.84, 5.35, 13.85, and 2.15%, respectively, in the samples studied. The combination of FTIR spectra with chemometric methods such as PCA and PLS-DA was used to distinguish the three types of betel. Discriminant analysis can classify black betel (*Piper acre*), *Piper betel*, and *Piper crocatum* according to its type. These methods can be used for identification and authentication of black betel.

## 1. Introduction

Black betel (*Piper acre* Blume) is a type of plant widely used in traditional medicine in Indonesia, especially in East Kalimantan. There are also various other types of betel in Indonesia, namely *Piper betle* and red betel (*Piper ornatum*). These three types of betel have the same genus name but are different varieties, and the types of compounds in each of the plants are relatively similar [1,2,3]. Traditionally, the types of betel plants were distinguished through their distinctive aroma, leaf shape, and spicy chelate taste. The differences indicate the levels of certain compounds found in the betel plants that are correlated with the resulting biological activity [2].

**Figure 1:**
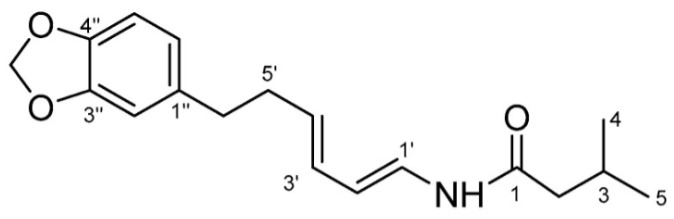
Structure of the piperenamide A compound [4].

The people of East Kalimantan have deep-rooted traditions and knowledge regarding the use of medicinal plants, which are still practiced today [2]. *Piper acre* Blume, as a traditional East Kalimantan medicine, has been commonly used for its properties to suppress fever, remove dampness, eliminate toxicity, and for its antimicrobial properties for centuries. In modern phytochemical and pharmacological studies, the alkaloids have recently been reported to have significant anti-viral, anti-inflammatory, anti-cancer and antimicrobial properties [2]. Black betel leaf ethanol extract contains piperenamide A (Figure 1), which is a new alkaloid, with antimicrobial properties [2,4]. A previous study has reported that the ethanol extract of black betel leaf contains the compound piperenamide A, which has increasing inhibition activity with increasing concentrations of black betel leaf ethanol extract on the growth of *S. mutans*, *S. sanguinis*, and *Candida albicans* [4].

The substitution of raw materials for a herbal medicinal product with closely related plants can be a serious problem because it will result in inconsistent efficacy. Empirically, the people of East Kalimantan believe that the efficacy of black betel is greater than other types of betel. This was followed by the results of a study that stated that the acquisition of IC_50_ values for the antioxidant activity of black betel ethanol extract is higher than that of green and red betel leaf ethanol extracts, namely 8.123 µg/mL [5]. The price for black betel is generally higher, and its availability lower, than *Piper betel* and red betel, raising concerns about the possibility of contamination and adulteration [6,7,8]. This situation can be the reason why green betel or red betel may be a potential forgery of black betel. It is also difficult to distinguish between the three types of betel if they are traded in the form of simplicia or powder since they are similar to one another. Several studies have reported the adulteration of species, ranging from 20% to 80% of raw herbal samples, which greatly impacts the biological activity and chemical composition of the ingredients [6,8,9,10]. Therefore, identification and discrimination of black betel from other types of betel plant is very important in ensuring authenticity, quality, safety, and efficacy before it is converted into the final product [10].

The common method for identification and authentication to control the quality of raw materials or plant extracts is by identifying the levels of one or several active compounds, known as the identifying compound approach [11]. Approaches based on marker compounds have several constraints, such as the number of marker compounds or active compounds that are limited and often not unique. In addition, the efficacy effect is not necessarily only influenced by a single compound because it is possible that there is a synergistic effect. The second approach is using a multicomponent approach, such as fingerprint spectral patterns, which are currently used for quality control of herbal medicinal raw materials [12]. The combination of these two approaches can be used because chemically herbal medicine is a multicomponent system. For the purposes of quality control, a fingerprint spectrum pattern can provide more accurate and realistic information. *Piper acre* Blume has been chemically studied and identified for the presence of sesquiterpenes, piperenamides, and isobutylamides [4,12]. Piperenamide A has been isolated from the leaves of *Piper acre* Blume, and exhibits important antibacterial activity [4].

Several analytical techniques such as chromatography (TLC, HPLC, and GC) and spectroscopy (UV-Vis, FTIR, and NMR) can be used for the purpose of identification, discrimination, and authentication of plants in order to guarantee the quality of raw materials. Among these techniques, the combination of analytical chromatography and spectroscopy is an attractive choice because it can meet the criteria for efficient analysis, such as being easy to use, fast, and inexpensive [13]. The resulting combination of data produces very complex information that thoroughly describes the characteristics of a sample.

H. Vogel et al. identified four species of medicinal herbs, bailahuén-species, *Haplopappus remyanus*, *Haplopappus multifolius*, and *Haplopappus taedaare*, using TLC resin [14]. The HPLC analytical technique is suggested for good *Wuweizi* quality control [15]. Wan et al., in 2022, categorized various parts of *Camellia nitidissima* samples using FT-IR with a combination of principal component chemometrics (PCA) and PLS-DA [16].

The FTIR spectrum can be used to distinguish one plant from another, even though the composition of the chemical compounds is not known with certainty [17]. The complex pattern of the IR spectrum makes direct and visual interpretation difficult. To make it easier, chemometric techniques such as multivariate analysis are needed [18]. The advantage of using chemometric techniques for interpretation of IR spectra is the ability to relate spectral profiles to hidden information contained in the sample [19]. There have been many studies that have combined the use of FTIR fingerprint spectra with chemometrics with the aim of identification, authentication, and discrimination of closely related plants [20,21,22,23,24].

Until now, no study has been performed on the authentication of *Piper betel*. Based on the above background, developing analytical methods for quality control (identification and authentication) of black betel using the analytical techniques of HPLC chromatography and FTIR spectroscopy in combination with chemometrics is important. Therefore, in this study, the development of an analytical method using a combination of chromatography and FTIR on black betel, green betel, and red betel, obtained in East Kalimantan was carried out. Black betel leaf samples were taken from five different locations in the East Kalimantan Region and extracted with 70% ethanol solvent, and HPLC analysis was performed on the ethanol extract. A combination of FTIR and chemometrics was used to determine whether piperenamide A can be used as a marker compound in the ethanol extract of black betel leaves.

## 2. Results

**Figure 2:**
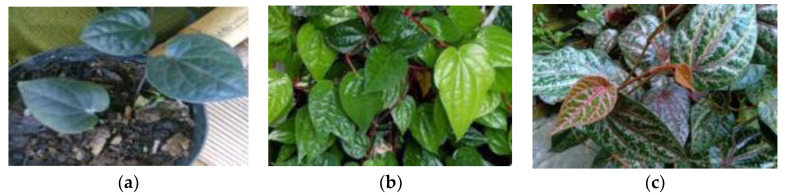
Types of betel plants: (**a**) *Piper acre* Blume; (**b**) *Piper betle* L.; and (**c**) *Piper crocatum*.

The black betel (*Piper acre* Blume), *Piper betle*, and red betel (*Piper crocatum*) plants descriptively have similarities and differences that are visible visually (Figure 2).

**Figure 3:**
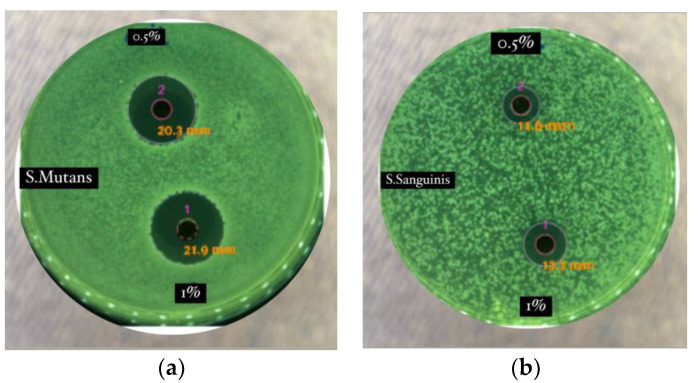
Antimicrobial activity of *Piper betle* var. *nigra* extract. (**a**) Antibacterial activity against *Streptococcus mutans*; and (**b**) antibacterial activity against *Streptococcus sanguinis.* 1 (concentration of 1%); 2 (concentration of 0.5%) [4].

It has been reported that alkaloids, terpenoids, flavonoids, polyphenols, tannins, and saponins were identified from black betel extract [4]. Flavonoids, polyphenols, and tannins are known to have antibacterial activity, with at least five possible mechanisms: damaging cell membrane permeability, inhibiting protein synthesis, damaging bacterial cell walls, inhibiting ATP synthesis, and disrupting cells [4]. The antibacterial activity in this experiment was postulated to be from the activity of piperenamide A isolated from black betel extract [4], shown in Figure 3.

### 2.1. Identification and Standardization

In this study, three different extract samples were used, namely black betel extract, green betel extract, and red betel extract. Black betel was obtained from five different locations (Table 1), with weights of 34.11, 40.5, 39.6, 51.57, and 21.17 g, respectively. The yield of black betel extract (*Piper acre* Blume) from each sample was 5.30, 6.07, 6.28, 8.17, and 3.14%, respectively. *Piper betel* extract was obtained from the location of East Kalimantan, with an extract of 70.8 g and yield of 14.16%. Red betel leaf condensed extract was obtained from the location of East Kalimantan, with an extract of 69.3 g and a yield of 13.86%.

The identification results showed that the samples were black betel, with the scientific name *Piper acre* Blume, green betel (*Piper bettle*), and red betel (*Piper ornatum*), belonging to the piperaceae family. In this study, the part of the plant used was the leaf (folium). The organoleptic parameters of black betel extract are blackish brown in color, characteristic odor, thick consistency, bitter taste, and slightly spicy and distinctive. *Piper betle* L. extract has the organoleptic properties of a thick, dark green color, and sharp betel odor. Red betel ethanol extract has the organoleptic properties of a thick, dark green color, very bitter taste, and distinctive betel smell.

The level of water-soluble compounds in the ethanol extract of black betel leaves averaged 12.08 ± 0.46%, meeting the quality requirements because its content is greater than 6% based on the requirements through the family approach in SNI 06-3953-1995. The drying shrinkage of the extract was an average of 8.89 ± 1.33%. For the drying shrinkage parameter, there are no terms or ranges of values used. The results of the total ash content in the extract was an average of 6.13 ± 1.07%. The results of the acid insoluble ash content in the extract was an average of 2.79 ± 1.56%.

### 2.2. Quantitative Analysis Using High-Performance Liquid Chromatography

Accuracy studies were performed using concentrations of 40, 70 and 100 ppm, and precision studies were carried out using a concentration of 70 ppm. LOD and LOQ measurements were obtained from accuracy study data, namely at concentrations of 40, 70 and 100 ppm.

The HPLC method was validated according to the International Conference on Harmonization (ICH-Q2(R1)) [25,26,27], with the results shown in Table 2.

**Figure 4:**
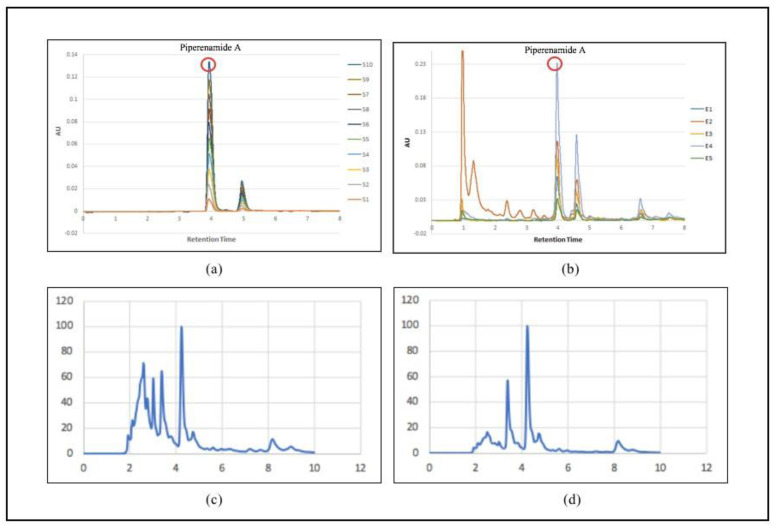
Chromatographic separation of (**a**) standard piperenamide A; (**b**) samples of black betel extract; (**c**) *Piper betel* extract samples; and (**d**) samples of red betel extract using a Purospher RP−18 column under a gradient elution of water and acetonitrile.

Figure 4a shows the piperenamide standard chromatogram profile at a concentration of 10–100 ppm. S1 = concentration of 10 ppm; S2 = 20 ppm concentration; S3 = 30 ppm concentration; S4 = 40 ppm concentration; S5 = 50 ppm concentration; S6 = 60 ppm concentration; S7 = 70 ppm concentration; S8 = 80 ppm concentration; S9 = 90 ppm concentration; and S10 = concentration of 1000 ppm. Figure 4b shows the chromatogram profiles of five samples of black betel leaf ethanol extract. E1 = sample extract 1; E2 = sample extract 2; E3 = sample extract 3; E4 = sample extract 4; and E5 = sample extract 5. Piperenamide A was only found in samples of black betel ethanol extract with retention times of 3.968, 3.973, 3.959, 3.966, and 3.980 min. This chromatographic appearance was also shown in all samples of black betel ethanol extract. However, the profile of the compound piperenamide A was not obtained in the chromatographic images of the ethanol extract of *Piper betel* and ethanol extract of red betel.

All validation data in Table 2 showed that the method meets the validation criteria based on international standards. Therefore, this method was further used to determine piperenamide levels in standard piperenamide A and samples of black betel leaf extract, as shown in Table 3.

### 2.3. FTIR-Chemometric Analysis

The PCA results in Figure 4 are in the form of a plot showing the grouping of the FTIR spectrum results.

**Figure 5:**
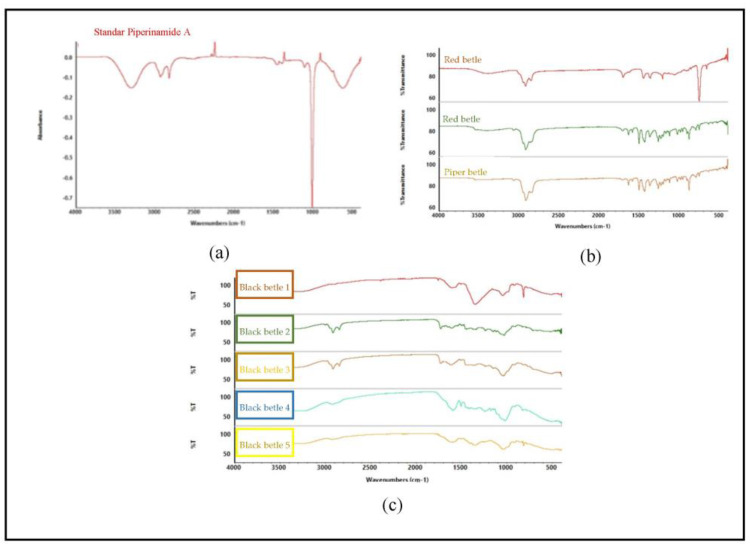
FTIR results of standard piperenamide A and the five samples. (**a**) FTIR spectra of piperenamide A; (**b**) FTIR spectra of samples of black betel leaf extract, green betel leaf extract, and red betel leaf extract; and (**c**) FTIR spectra of 5 samples of black betel extract from East Kalimantan.

The appearance of the spectrum affects the composition of the metabolites present in the sample. Figure 5b shows the spectra for different species but in the same family, indicating different metabolite content in each sample. Figure 5c shows that all samples contain piperenamide A, with different concentrations. The different concentrations of piperenamide A result in different spectra because, when the concentration is less, it indicates that there are other components present in the extracts, causing the spectra to become more visible and different from one another. The FTIR results were processed using PCA (Figure 5) to confirm these FTIR results.

**Figure 6:**
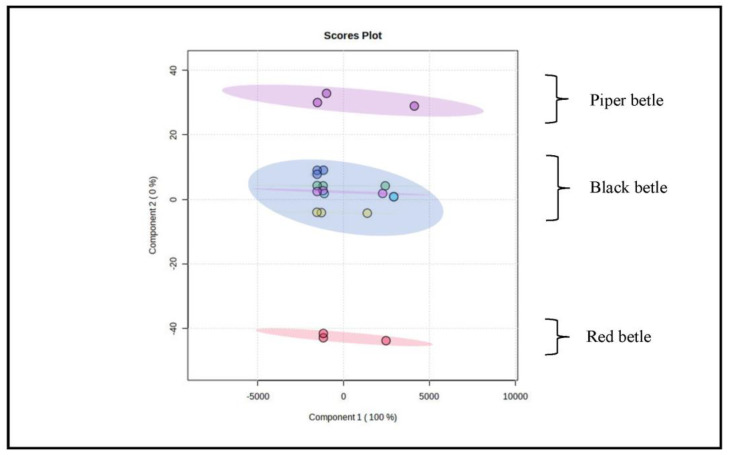
Overview of PCA chemometric results using MetaboAnalyst software 5.0. Annotation: purple circle: Piper betle sample; blue circle: Black betle sample; red circle: Red betle sample.

Figure 6 represent the results of the score plot display, indicating that the initial two-component plot can 100% explain the total variance. The pattern of grouping samples using PCA could not clearly distinguish all samples because there was still an accumulation of plots. These results also indicate that all the samples, especially those that are close together and even stacking, have similar characteristics to one another.

**Figure 7:**
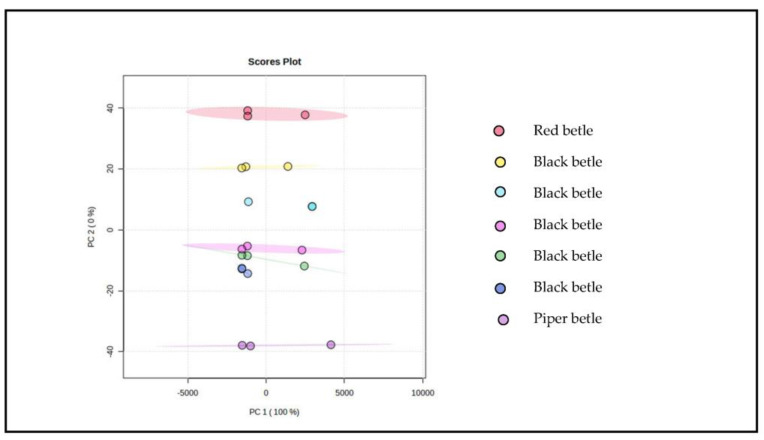
Overview of PLS−DA chemometric results using MetaboAnalyst software 5.0.

Then the component values obtained from PCA were used to build a model for identifying and authenticating black betel. This is performed because the characteristics of the FTIR spectrum of each sample have a different correlation in each sample. Interestingly, a 100% diversity of data is obtained, as shown in Figure 7, indicating the discriminant function obtained can distinguish the three types of samples and even distinguish the same types of samples based on the characteristics of the main components used in the modeling.

## 3. Discussion

*Piper betel* is a type of climbing herbaceous plant. The leaf shape is flat, resembling a heart, the stem is rather long, the leaf edge is flat, the leaf tip is tapered, the leaf base is notched, the leaf bones are pinnate, and the leaf flesh is thin. The leaf surface is green and smooth, while the tree trunk is slightly brownish green and the skin surface is rough and knuckle [28]. Black betel (*Piper acre* Blume) and red betel (*Piper crocatum*) have the same shape and characteristics as *piper betel* herbarium. The differences are that the black betel leaves tend to be smaller and thicker, and the leaves and petioles are darker or black in color. Meanwhile, red betel is often used as an ornamental plant because of its attractive appearance. Red betel also has the same shape as *piper betel*, but the difference is that it has a distinctive color, namely the surface of the leaves is dark green combined with red-heart veins, and heart-purple mixed with silver, and is shiny [29].

The maceration results of each sample were evaporated with a rotary evaporator to obtain a thick extract. The ethanol extract of black betel leaves was standardized with two parameters, namely specific parameters and non-specific parameters. Specific parameters include identity, organoleptic properties, water-soluble compound content, and chemical content, while the non-specific parameters include drying shrinkage, total ash content, and acid-insoluble ash content. The ethanol extracts of *piper betel* and red betel were only subjected to organoleptic tests as specific parameters.

It has been reported that the ethanol extract of black betel has antibacterial activity against two pathogenic bacteria in the oral cavity. Black betel extract at all concentrations from 0.5% to 1% inhibited the growth of *Streptococcus Mutans* and *Streptococcus Sanguinis*, with increased inhibition diameters with increasing concentrations of extract (Figure 2). *Streptococcus mutans* was more sensitive compared with *Streptococcus sanguinis* to the ethanol extract of black betel (0.5%), with inhibition diameters of 18.2 mm and 9.9 mm, respectively. This shows that the activity of piperenamide A derived from the black betel plant has considerable potential as an antibacterial candidate for herbal medicinal raw materials [4].

The similarity between closely related species such as black betel, *Piper betel*, and red betel is feared to be an opportunity for drug counterfeiting. The development of analytical methods for the identification and authentication of black betel is crucial, considering its potential as a raw material for herbal medicine, before being used in commercial products. Currently, the combination of chromatographic, spectroscopic, and chemometric analysis is a good choice for this purpose. In this study, method validation was first carried out, followed by chromatographic analysis using HPLC to obtain the levels of the compound piperenamide A. Principal component analysis (PCA) and discriminant analysis (PLS-DA) of the FTIR spectrum were then carried out to identify and authenticate black betel from *Piper betel* and red betel.

Parameter validation met the requirements set by Q2(R1)-ICH (International Conference on Harmonization) [25,26,27]. Method validation for the analysis of piperenamide A compounds produced valid data. The results of the parameters used show that they can be applied to the routine analysis of piperenamide A raw material and preparation products.

The analysis was then continued using a validated HPLC system to identify and measure the content of piperenamide A. Piperenamide A was found only in samples of betel black ethanol extract with a retention time of 3.9 min in all samples (Table 2). The results of quantitative analysis of each sample of the ethanol extract of black betel leaf (*Piper acre* Blume) obtained from five different locations in the East Kalimantan Region showed the presence of piperenamide A at values of 4.03; 6.84; 5.35; 13.85 and 2.15%, respectively, in the samples. Based on Table 3, the highest content of piperenamide A was in sample code E4, originating from Samarinda City.

FTIR spectroscopy is a fast, simple, and non-destructive analytical technique in which all chemical properties in a sample can be expressed and displayed in the FTIR spectrum. Figure 5 shows that there are differences in the spectra of the three types of betel. The spectra between piperenamide A and the five samples of black betel leaf ethanol extract have different spectrum characteristics. The FTIR spectral profiles of the five ethanol extracts of black betel leaves used demonstrate different patterns because the components and/or the magnitudes of the components contained therein are different. The results obtained from the analysis showed that each sample had similarities in term of the presence of the piperenamide A compound, but the levels were different [30].

The IR spectrum shows bands originating from amines (Vmax 3295 cm^−1^), amide carbonyls (Vmax 1656 cm^−1^), isolated conjugated double bonds (Vmax 1611 and 1504 cm^−1^), and ether groups (Vmax 1141 cm^−1^). This identical spectrum pattern causes data interpretation to be difficult so it is necessary to add chemometric techniques such as multivariate analysis [18]. Principal component analysis (PCA) is a chemometric method used to classify the properties of materials or substances based on their similarities [31]. Chemometric analysis was performed with MetaboAnalyst software 5.0 using the principal component analysis (PCA) method.

From the PCA results (Figure 6), it can be seen that the angles between all samples are close to each other and some even overlap. Score plot analysis provides an overview of the relationship between variables and relative similarity between observation objects by examining the distance between variables and samples. The closer the distance between the two variable points and the sample is, the greater is the variable that contributes to the sample. The results of the score plot displayed in Figure 6 illustrate that all points are close to one another and the characteristics of all samples are relatively close [32].

Grouping on the score plot is a function to determine the similarity of the characteristics of each sample [32]. Figure 6 shows that the main components are able to explain the variance of the data by 100%. The results of the description of the score plot show that the grouping was successful based on the close response values. The characteristics between the groups were almost the same and the response values were not much different. These results also show that there are groups of samples with response values close together and, even if the buildup occurs, they have chemical characteristics that are very similar to one another [32,33,34,35].

The sample grouping pattern using PCA (Figure 6) has response values that are not scattered. These results also show the importance of developing PLS-DA modeling to strengthen the data as characterization compounds. The modeling on the PLS-DA data settings is governed by the selected principal component analysis obtained from the FTIR spectrum, with three data repetitions for each sample, which will be the PLS factor. The PLS factor is an important parameter for assessing the quality of the model, and this factor indicates the level of complexity and acceptability of the model setting data in the software used [36]. In general, the number of PLS factors involved in a PLS model is highly dependent on the range of the wavelength spectrum and modeling techniques [36].

The results of PLS-DA (Figure 7) show a good quality model with a value of R^2^ = 0.81. Modeling shows the relationship between the response variables category and predictor variables that are numerical in nature and, in this case, characterizing compounds [37,38,39]. Hence, the modeling succeeded in differentiating the ethanol extract of black betel from the ethanol extract of green and red betel. In addition, the combination modeling was also able to identify piperenamide A compounds based on the number of levels in different samples. Therefore, piperenamide A can be considered as a characterization compound for black betel [32,33,34,35].

## 4. Materials and Methods

### 4.1. Plant Material

*Piper betel* and red betel leaves were collected randomly from East Kalimantan. Black betel leaves were collected from 5 different growing locations in East Kalimantan, Indonesia. The plant was later identified as BRIN, Bogor, Indonesia, with data number 2086/IPH.1.02/II.8/XII/2018.

### 4.2. Extraction

The extraction method was adapted from Junairiah et al. (2018), where the acquisition of each sample based on the location of growth is given the same treatment. Dried leaves were extracted with 70% ethanol at room temperature for 3 days. After that, each sample was removed using a rotary evaporator to obtain a thick ethanol extract [40].

### 4.3. Materials

NB (liquid media) (NB; Oxoid, Hampshire, UK), MHA (solid media) (MHA; Oxoid, Hampshire, UK), acetonitrile HPLC grade (Merck), water (Milli-Q pure water from an Arium^®^ Pro), piperenamide A used as a standard is an isolated compound with a purity of 70–80%, Waters 2695 brand HPLC set (Milford, MA, USA), brand analytical balance, micropipettes, and glassware. FTIR spectrometer set of Thermo Scientific (Waltham, MA, USA) type Nicolet Summit FTIR Spectrometer Everest ATR Diamond, and MetaboAnalyst software 5.0.

### 4.4. Antimicrobial Activity

The antimicrobial activity of *P. betle* extract was studied using the well diffusion method against two oral pathogenic bacteria (*Streptococcus mutans* and *Streptococcus sanguinis*). Bacterial suspension inoculums were prepared from 24 h cultures on NB for bacterial suspensions. The inoculum was diluted with a sterile physiological solution (0.9%) to 108 CFU/mL (McFarland standard 0.5). Then, 20 mL of each agar medium was thawed, cooled to 50 °C and then inoculated with 0.2 mL of the microbial suspension. Inoculated agar was poured into sterile petri dishes, then allowed to cool on a flat surface. After the medium had solidified, two wells were cut from the agar, each 6 mm in diameter. Then, a 30 µL sample extract (0.5 and 1% concentrations) was added to each well and incubated for 24 h at 36 °C ± 1 °C under aerobic conditions. Microbial growth inhibition was measured in mm using a SCAN 500^®^ tool. The test was carried out in duplicate.

### 4.5. Method of HPLC Preparation

The ethanol extracts of black betel, *Piper betel*, and red betel leaves were weighed (0.5 mg each) using an analytical balance into a 10 mL volumetric flask and acetonitrile was added up to the mark, then the mixture was sonicated. After that, the solution was filtered using 0.45 µm membrane filter paper (Whatman), then 10 µL of the filtrate was taken to be injected into the HPLC device.

For HPLC operational conditions, HPLC brand Waters 2695 is equipped with an auto sampler and a photodiode array detector, with the following conditions: injection volume 10 µL, flow rate 1.0 mL/minute, wavelength 259.2 nm, and column: LiChroCART, 250 mm^–4^, 6 µm (Purospher RP-18, 5 µm). The mobile phase used is a gradient system (water:acetonitrile). In the initial HPLC conditions for the mobile phase, a mixture of water and acetonitrile (50:50) is used: in the 1st minute the mobile phase is fixed with a mixture of water and acetonitrile (50:50), then in the 2nd minute the mobile phase is a mixture of water and acetonitrile (10:90) until the 8th minute, which is the end time of analysis.

This condition was repeated according to the program that was set up in the HPLC program settings used for HPLC chromatographic analysis of five samples of black betel ethanol extract, green betel ethanol extract, red betel ethanol extract and standard piperenamide A, for method validation and determination of compound levels in the samples. The validation of the test method was carried out with several test parameters, namely linearity, accuracy, precision, and LOD/LOQ.

### 4.6. Method of FTIR-Chemometric Preparation

All samples (extracts of black betel, *Piper betel*, and red betel) and standard piperenamide A were prepared, and then a small sample was placed thinly on the sample area evenly. One sample was scanned with 3 repetitions in the infrared region (4000–400 cm^−1^). This step was repeated for each of the other samples as well as the piperenamide standard. Furthermore, the wave number absorbance data obtained were processed with software and used to make a model for identifying and authenticating black betel from *Piper betel* and red betel. The multivariate analyses used were principal component analysis (PCA) and discriminant analysis (PLS-DA). The software used in modeling was MetaboAnalyst version 2012 (Addinsoft, New York, NY, USA).

## 5. Conclusions

Based on the results, it can be concluded that the ethanol extract of black betel (*Piper acre* Blume) contains piperenamide A. The extract code E4 from Samarinda City has the highest levels of piperenamide A. PLS-DA chemometric analysis shows an R^2^ value of 0.81, indicating that the combination of FTIR spectra with chemometric methods such as PCA and PLSDA is able to distinguish the three types of betel. Discriminant analysis can classify the three types of betel according to type. This method can be used for identification and authentication of black betel.

## Figures and Tables

**Table 1:** Sampling locations.

No.	Betel Type	Sample Code	Source
1.	Black Betel	E1	Balikpapan
2.		E2	Kutai Kartanegara
3.		E3	Berau
4.		E4	Samarinda
5.		E5	Kutai Timur
6.	Green Betel	S6	Samarinda
7.	Red Betel	S7	Samarinda

**Table 2:** Parameter validation results.

Validation Parameters	Value Obtained	Reference
Linearity	r = 0.998	r ≥ 0.998
Accuracy	% recovery = 100.34%	98–102%
Precision	% RSD = 1.04	% RSD ≤ 2
LOD	1.32	-
LOQ	4.01	-

**Table 3:** Levels of piperenamide A.

Sample	Content (%)
E1	4.03 ± 0.04
E2	6.84 ± 0.20
E3	5.35 ± 0.12
E4	13.85 ± 0.21
E5	2.15 ± 0.13

## Data Availability

Data are contained within the article.

## Sample Availability

Samples of black betel leaf extract are available from the correspondence author.
